# Whole‐Genome Sequencing‐Based Monitoring of *Cronobacter sakazakii* ST4 Persistence in an Irish Dairy Powder Facility

**DOI:** 10.1002/fsn3.71717

**Published:** 2026-05-15

**Authors:** Qicheng Hao, Francis Butler

**Affiliations:** ^1^ UCD School of Biosystems and Food Engineering University College Dublin Dublin Ireland

**Keywords:** *Cronobacter sakazakii*, powdered infant formula (PIF), whole‐genome sequencing (WGS)

## Abstract

*Cronobacter sakazakii,* an emerging foodborne pathogen linked to neonatal infections, poses significant risks in dairy powder processing facilities, particularly those supplying ingredients for powdered infant formula (PIF). This case study employed whole‐genome sequencing (WGS) to monitor *C. sakazakii* persistence in a large‐scale Irish high‐protein milk powder plant over a 12‐month period, focusing on ultrafiltration, evaporation, spray‐drying, and packaging zones. Environmental swab samples of 240 in total from 20 fixed locations were collected monthly and analyzed using the ISO 22964 method, followed by real‐time PCR confirmation and Illumina‐based WGS for isolate characterization. Eighty‐eight *Cronobacter spp* positive samples were isolated out. Multilocus sequence typing identified four sequence types, with 10 isolates of ST4 associated with clinical meningitis. Phylogenetic analysis revealed two distinct ST4 clades: one persistent but spatially limited (isolates K08 and C19), detected sporadically across months, and another clonal group exhibiting temporal proliferation, peaking in the final month across multiple dryer levels. These findings indicate recurrent contamination events and long‐term persistence, underscoring WGS's superior resolution over traditional typing methods for tracking pathogen dynamics. The method of WGS, as the new gold standard, offers the highest resolution, the greatest amount of information, permanent traceability, lower cost, and faster speed in bacterial analysis—advantages that MLST (Multilocus sequence typing) cannot match. The study demonstrates WGS as a powerful tool for enhancing surveillance in dairy facilities, enabling early detection of colonization risks and informing targeted hygiene interventions to safeguard PIF supply chains. Therefore, the rational and widespread adoption of WGS in food safety surveillance can markedly simplify and accelerate the precise identification and source tracking of contaminants, thereby providing robust technical support for the early prevention and rapid response to food safety incidents.

## Graphical abstract

In dairy powder manufacturing facilities, the repeated isolation of clonally related *Cronobacter sakazakii* isolates underscores the persistent challenge of eliminating contamination risks posed by this pathogen, which may rapidly proliferate under conducive environmental conditions. Whole‐genome sequencing (WGS), offering superior discriminatory resolution, provides a robust technical framework for precise monitoring and source tracking within PIF production environments. Schematic overview of *C. sakazakii* contamination monitoring and source tracking in dairy powder production environments using whole‐genome sequencing.

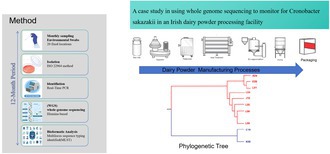

## 1 Introduction

*Cronobacter* spp. is commonly associated with powdered infant formula. The bacterium is regarded as an emerging foodborne pathogen leading to several outbreaks as reviewed by Singh et al. (2015). Numerous cases of *Cronobacter sakazakii* infant infection have been documented (Mullane et al. 2007). *Cronobacter* spp. has frequently been associated with powdered infant formula production facilities (Mullane et al. 2007). In recent years, the PIF industry has made significant efforts to control *C. sakazakii* in their production facilities. Mitigation strategies have included enhanced raw ingredient, environmental and product monitoring, and the introduction of dry cleaning programs in the production areas to minimize wet environments that allow *C. sakazakii* the opportunity to proliferate. Enhanced raw ingredient monitoring for *C. sakazakii* has led to an increased focus on *C. sakazakii* surveillance in dairy process facilities manufacturing dairy powders and ingredients that potentially are used in PIF production. The occurrence of *C. sakazakii* has been reported in other dairy powder manufacturing facilities. Von Westerholt (2018) undertook surveillance work on *C. sakazakii* in a dairy ingredient manufacturing facility and isolated some strains with genotype ST4 and ST99. In addition, anecdotally at least, dairy ingredients companies have also begun to use *Cronobacter* spp. as an additional indicator organism in their overall environmental hygiene monitoring programs to supplement more traditional indicators, such as coliforms or Enterobacteriaceae. Despite these extensive control measures, *C. sakazakii* is frequently reported as a resident contaminant in dry dairy processing environments, where it can persist for months to years due to its exceptional tolerance to desiccation and ability to form biofilms on surfaces such as floors, equipment, and air‐handling systems (Tong et al. 2024). Of particular concern is sequence type ST4, which is the predominant clinical lineage globally and is strongly associated with severe neonatal meningitis and necrotizing enterocolitis, exhibiting significantly higher invasiveness and case‐fatality rates compared to other STs (Forsythe et al. 2014). The repeated isolation of ST4 and other genotypes from dairy ingredient facilities (Von Westerholt 2018) underscores the ongoing risk of post‐pasteurization recontamination, even in products not destined for infant formula.

The heat resistance of *Cronobacter* spp. is not high enough for it to survive pasteurization, so contamination of dairy ingredients most likely occurs during the final drying of the powder and packaging. When raw milk arrives at a dairy ingredient factory, milk is pasteurized using HTST treatment by heating to at least 72°C and holding at or above this temperature for at least 15 s, which is the pasteurization process to kill most bacteria including *Cronobacter* spp. The next step of the manufacture is concentration and spray drying. Spray drying involves atomizing milk into a hot air stream (180°C–220°C). The size of the droplets, the air temperature, and the airflow can be controlled in the spray dryer. The primary aim of this step is not to use high temperature to inactivate bacteria, so the possibility of *Cronobacter* spp. survival should be considered. Given that pasteurization effectively eliminates *Cronobacter* spp. from raw milk, contamination of final powder products almost exclusively originates from the dry processing environment post‐heat treatment. The low water activity and periodic dry‐cleaning practices in these zones create ecological niches that favor the long‐term persistence of desiccation‐resistant *Cronobacter* populations (Tong et al. 2024). This means that if *Cronobacter* spp. are present in the environment of the production areas after the pasteurization process, it may contaminate the final products. Environmental monitoring for *Cronobacter* spp. in process areas is essential as an early warning for the potential presence of *Cronobacter* spp. in powder products.

The objective of this work was to undertake an extended surveillance program for *Cronobacter* spp. in the environment of a large‐scale Irish high protein milk powder processing facility as a case study in using whole‐genome sequencing as a fingerprinting tool for monitoring for bacterial pathogens in a dairy process facility environment. The milk powder was not destined as an ingredient in infant formula or foods intended for special medical purposes. Surveillance included environmental swabs taken monthly from the part of the facility housing the ultrafiltration process, evaporation, spray‐drying, and powder packaging. Surveillance continued over a 1‐year period to capture the seasonal change in occurrence in *Cronobacter* spp. within the environment. Although whole‐genome sequencing (WGS) has become a powerful tool for outbreak source‐tracking, its application in long‐term (≥ 12 months), high‐resolution longitudinal monitoring of *Cronobacter* within a single manufacturing facility to elucidate clonal persistence and transmission dynamics remains rare (Mousavi et al. 2023). Next generation sequencing was used to identify and characterize all positive *Cronobacter* spp. isolates recovered from the facility to allow precise identification of the isolates recovered from the process environment. When coupled with other spatial and temporal meta‐data collected, the genomic data was used to identify potential sources of contamination and persistence of the pathogen in the process environment.

## 2 Materials and Methods

### 2.1 Milk Proteins Concentrate Production Facility

The process facility studied was a large Irish dairy plant manufacturing high protein milk powder. This powder was not intended for infant formula manufacture. This study concentrated on the part of the process environment housing concentration and final spray drying of the powder. Surveillance of the process environment in the facility was taken over a 12‐month period. Twenty sampling locations were selected and were sampled monthly. Among the 20 sampling locations, locations 1–7 were in the wet processing area, where daily wet cleaning was performed, and the surfaces were heat‐, water‐, and chemical‐resistant materials. The remaining locations (8–20) were in the dry processing area, where regular dry cleaning was used. In the dry area, floors were made of epoxy resin, hatches on the tower and similar equipment surfaces were polished stainless steel, and tools such as brushes and shovels were food‐grade plastic. All samples were collected at ambient temperature. In total, 240 samples were collected. Table 1 sets out the sampling locations selected. The locations include equipment surfaces; walls, fixtures, and floors; equipment such as brushes and shovels used by operators in the facility.

**TABLE 1:** *Cronobacter sakazakii* sampling locations within the process facility.

Code	Sample point
	**Drier Level 1**
1	Transition Zone Crossover Bench
2	Sifter Unit
3	Air Louvre
4	Sifter t
	**Drier Level 2**
5	Fan
6	Hatches on drier
7	Vacuum Cleaner
	**Drier Level 3**
8	Hatches on Tower
9	Floor at Entry Door
10	Power Port on Wall
	**Drier Level 5**
11	Steps to Atomizer
12	Brush & Shovel
13	Floor at Entry Door
	**Concentration AREA**
14	Floor Area 1
15	Floor Area 2
	**Bagging Sieve Area**
16	Service Port Floor
17	Floor at Wall
18	Power Cable on Wall
19	Ledge over Entry point
20	Entrance door

### 2.2 Environmental Sampling

**FIGURE 1:**
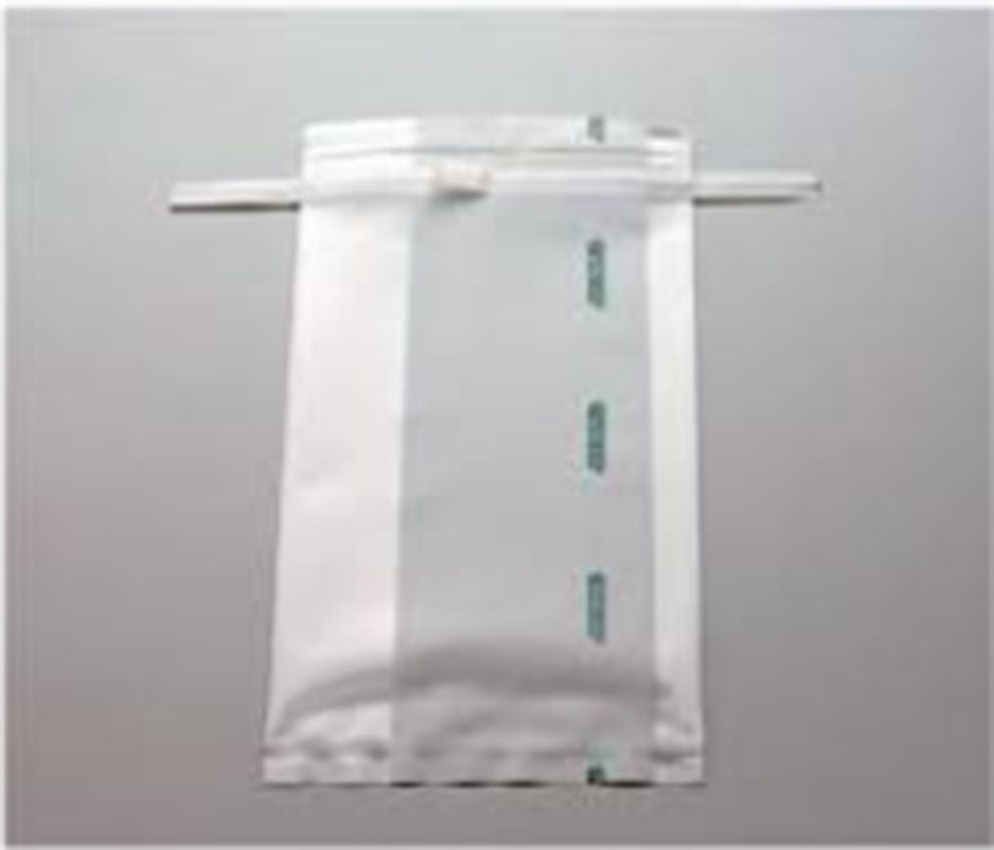
An example of the TWIRL'EM sterile bag for swab sample collection (for illustration only).

Sterile Swabs (Sani‐Stick kit, Labplas, Quebec) were used to swab the surface of the 20 locations. The Sani‐Stick is a sterile sponge with an integrated handle which comes in a TWIRL'EM sterile sampling bag (Figure 1). After swabbing, the handle is released, and the sponge was put into a 120 mL sterile bag and sealed. Quality Control staff from the manufacturing facility took the swab samples using sterile templates to delineate the swabbing area. The templates were 10 cm × 10 cm. The sample swabbing technique was three strokes down, three strokes diagonally, and three strokes from left to right. Subsequent repeat samples were sampled from the same approximate location as set out in Table 1. The swabs were refrigerated at 4°C immediately after swabbing. The following day, the swabs were transferred to University College Dublin in an icebox for analysis and stored overnight at 4°C before microbiological analysis the following day.

### 2.3 Cronobacter spp. Determination

The following day after receipt of samples, the samples were tested for *Cronobacter* spp. in accordance with ISO 22964, (ISO 2017). The sponge was transferred into a 280 mL stomacher bag, then 90 mL buffered peptone water (CM0509, Oxoid) was added into the bag and stomached for 2 min. The liquid was poured into a sterile 120 mL tube. This was then incubated for 18–24 h at 37°C. Hundred microliter of liquid was then inoculated into 10 mL *Cronobacter* Screening Broth (CM1121, Oxoid) which was supplemented with one vial of Vancomycin Supplement (5 mg) (SR0247E, Oxoid) and then incubated at 42°C for 18–24 h. A color change from purple to yellow was indicative of the presence of *Cronobacter* spp., that is, presumptive positive result. For presumptive positive samples, 10 μL of the positive sample was streaked onto ESIA ( *Enterobacter sakazakii* isolation agar) (CM1134, Oxoid) by 10 μL inoculating loop and incubated for 18–24 h at 37°C. A blank plate was used as the negative control and *C. sakazakii* strain ATCC 29544 (GenBank FR714908.1) was used as the positive control. A positive result was indicated by blue colonies. Eighty‐eight of 240 samples were showed as *Cronobacter* spp‐positive blue.

For positive samples, one colony was picked from the ESIA plate and streaked onto Tryptic Soya agar and then incubated at 37°C for 10–24 h. The colonies were re‐suspended from the TSA plate into Tryptic Soya broth supplemented with 20% glycerol for cryogenic storage. The samples were stored at −20°C for further confirmation using molecular analysis.

### 2.4 Real‐Time PCR Confirmation of Cronobacter spp.

To confirm the isolation result, real‐time PCR was used. A positive colony was picked from the ESIA or TSA plate, or from the overnight cultures on TSA as appropriate. The colony was picked by a 1 μL inoculating loop and homogenized into a 1.5 mL centrifuge tube containing sterile 50 μL 18 MΩ water. After vortexing for DNA extraction, the tubes were boiled on a heating block at 90°C for 10 min. Then, the tubes were spun at 10,000 rpm for 2 min to sediment cellular debris. The RT‐PCR reagents were prepared. The primers used are shown in Table 2. The DNA extract was added to the RT‐PCR reagents and loaded onto a Rotorgene R‐3000 real‐time PCR cycler for analysis. An isolate was considered positive for *Cronobacter* spp. when the amplification plot showed a typical sigmoid curve and the quantification cycle (Cq) value was ≤ 35. (Drudy et al. 2006; Mullane et al. 2008; Seo and Brackett 2005).

**TABLE 2:** Primers and Probe use in PCR test for the detection of *Cronobacter* spp.

Oligo name	Sequence (5′ > 3′)	Tm (°C)	GC (%)
MMS forward	GGGATATTGTCCCCTGAAACAG	60	50
MMS reverse	CGAGAATAAGCCGCGCATT	59	53
MMS Probe (hydrolysis probe)	6FAM‐ACAGAGTAGTAGTTGTAGAGGCCGTGCTTCC‐TAMRA	66.2	52

### 2.5 Whole Generation Sequencing

#### 2.5.1 DNA Extraction and Quality Control

The Mo Bio Microbial DNA Isolation Kit (Qiagen Catalog#12224–250) was used to extract microbial DNA. The DNA extraction was carried out following the published Qiagen protocol (https://www.qiagen.com/us/products/discovery‐and‐translational‐research/dna‐rna‐purification/dna‐purification/microbial‐dna/dneasy‐ultraclean‐novipure‐microbial‐kits/?catno=12224‐250). Before sequencing, the quality of DNA was determined using both Nanodrop and Qubit methods. A Nanodrop ND‐1000 Spectrophotometer was used to test DNA concentration. A 2 μL sample was loaded on the spectrophotometer for analysis. A Qubit fluorometer 2.0 was also used to test the quality of DNA. Qubit dsDNA BR (Broad Range) Assay Kit were used. A 2 μL DNA sample was added to the diluted assay reagent and the concentration was then read on the Qubit fluorometer. DNA purity was assessed using the A260/A280 and A260/A230 ratios on the NanoDrop ND‐1000. Samples with A260/A280 ratios of 1.8–2.0 and A260/A230 ratios > 1.8 were considered acceptable. Final DNA concentrations used for library preparation ranged from 50 to 150 ng/μL (measured by Qubit dsDNA BR Assay), with a minimum total yield of 1 μg. Typical acceptable DNA concentrations for subsequent sequencing was approximately 100 ng/μL. SNP calling and phylogenetic analysis were conducted using the closed genome of *C. sakazakii* strain SP 291 (GenBank CP00491.1) as the reference strain.

#### 2.5.2 Genomic Sequencing

Extracted DNA samples were sent for commercial sequencing by Novogene PLC. (Cambridge, United Kingdom). Sequencing was carried out on an Illumina Hiseq PE150 with a nominal > 100× coverage.

### 2.6 Bioinformatics

#### 2.6.1 Quality Assessment and Trimming of Raw Reads

The quality of the raw reads was first checked by FastQC (“FastQC,” https://qubeshub.org/resources/fastqc). Trimmomactic (Bolger et al. 2014) was used to trim all low quality and adapter sections from the raw read data.

#### 2.6.2 Genome Assembly and Quality Assessment

After the raw read data was quality assessed and trimmed, the reads were assembled into contigs using SPAdes (Version 2.12.1) (Bankevich et al. 2012). The “careful” option was selected to minimize the number of mismatches and short indels. After assembly, genome assembly quality was assessed using QUAST (Gurevich et al. 2013). Assemblies were subsequently annotated using Prokka (version 1.2.4) (Seemann 2014). Assembly quality metrics generated by QUAST showed an average N50 of 187,452 bp (range 98,216–412,875 bp), a mean total assembly length of 4.38 ± 0.06 Mb, and an average of 28 ± 12 contigs per genome (range 12–67 contigs). All assemblies had ≥ 98.5% of reads mapped back to the final assembly.

#### 2.6.3 Species Identification and Profiling

Species identification was carried out using Kmerfinder (Clausen et al. 2018). MLST profiling was carried out using the DTU CGE MLST 1.8 tool (Larsen et al. 2012). The settings used were “*Cronobacter* spp.” for MLST configuration and “Illumina—paired end reads” for read type.

#### 2.6.4 Phylogenetic Analysis

The CSI Phylogeny 1.4 tool (Kaas et al. 2014) was used in this study. This tool constructs a phylogenetic tree based on SNP analysis in comparison to a reference genome. A reference genome for the analysis was chosen for the *C. sakazakii* sequence type 4 detected during the monitoring program. This study focused on the occurrence of ST4 isolates found during the surveillance due to its reported links to clinical cases. To obtain a high quality closed reference genome, one ST4 isolate was sequenced using Nanopore DNA sequencing (Oxford Nanopore Technologies). Nanopore sequencing allows the analysis of long DNA fragments by monitoring electrical current changes at a pore surface as the DNA fragments pass through a protein nanopore. Special large‐pore tips were used to inject unfragmented DNA into Nanopore flow cells, where each nanopore is connected to its own electrode, channel, and sensor chip, ultimately decoded by base calling algorithms to determine the sequence.

CSI Phylogeny 1.4 (Kaas et al. 2014) was run with the following parameters: minimum depth at SNP positions of 10×, minimum relative depth at SNP positions of 0.1, minimum distance between SNPs of 10 bp, minimum SNP quality of 25, minimum read mapping quality of 25, and minimum Z‐score of 1.96. Only SNPs located in positions covered by at least 90% of all isolates were considered for tree construction. A maximum‐likelihood phylogeny was inferred using FastTree under the GTR model with 1000 bootstrap replicates. A phylogenetic tree for all positive *C. sakazakii* is given Figure S1.

## 3 Results

**FIGURE 2:**
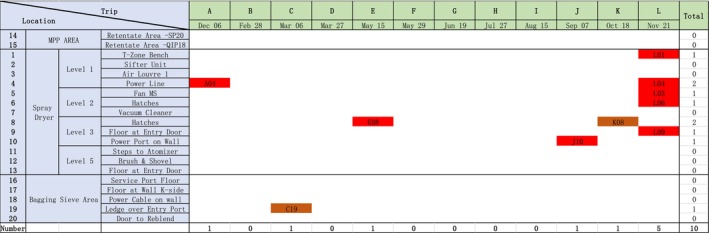
The surveillance *C. ronobacter sakazakii* sampling trips and locations of ST4.

**FIGURE 3:**
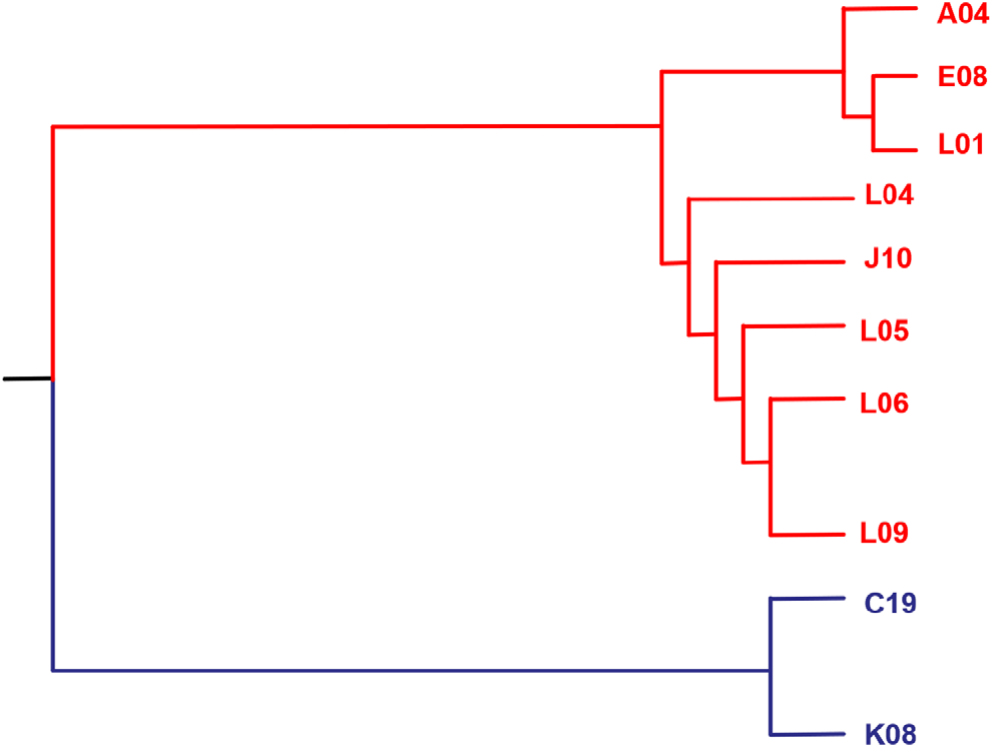
Phylogenetic tree for the *C. sakazakii* ST4 isolates recovered in the process facility(Snp differences shown in Figure 4).

**FIGURE 4:**
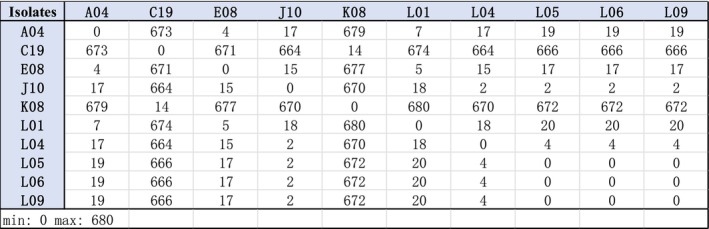
Distance Matrix for *C. sakazakii* ST4 isolates recovered in the dairy process facility.

Multilocus sequence typing (MLST) analysis using the DTU CGE MLST 1.8 tool indicated four sequence types, detected in the positive *C. sakazakii* samples. There were 10 positive samples of sequence type 4 detected during the monitoring period. Figure 2 illustrates the temporal and spatial distribution of the 10 positive *C. sakazakii* ST4 samples. From the phylogenetic tree for the 10 ST4 isolates (Figure 3), there was two distinct groups detected. Isolates K08 and C19 were very similar to each other (a SNP difference of 14 SNPs, Figure 4) and were quite dissimilar to the remaining eight isolates (a SNP difference of over 660 SNPs, Figure 4). Isolate C19 was detected in the bagging area in March, whereas the second isolate was detected in the spray dryer area in October, demonstrating that this particular strain while persisting, did not appear to be colonizing large areas of the process facility.

In contrast, the second group comprising the other eight ST4 isolates was very similar (a SNP difference of less than 20 SNPs, Figure 4) and would appear to be clonal and originated from the one contamination source. As Figure 4 shows, this strain was detected in the first month of monitoring (December) at level one in the spray dryer and was detected sporadically in May and September. However, in November, the strain was detected at five separate locations within the spray dryer, indicating a sudden proliferation in this particular strain. In summary, it would appear that there were two separate contamination events that introduced ST4 *C. sakazakii* strains into the process facility before/during the surveillance period that were then detected by the surveillance program.

## 4 Discussion

It is noteworthy that for one of the ST4 groups which was detected eight times in total, five of these detections were in the final monitoring period. It had also been detected in the initial monitoring period but only sporadically in the intervening period. The high level of detection in the final monitoring period could indicate that particular strain was finally beginning to colonize a broad area of the process facility. Whole‐genome sequencing (WGS) provided significantly higher resolution than previous methods such as pulsed‐field gel electrophoresis (PFGE) and multilocus sequence typing (MLST) used in earlier studies. This enhanced discriminatory power enabled the detection of low‐level persistence and potential recontamination events that might have been overlooked or interpreted as unrelated isolates with less precise typing techniques. In practical terms, these findings underscore the importance of targeted cleaning and environmental control measures, particularly in identified high‐risk areas or equipment niches, to disrupt persistent strains and prevent their reemergence. This type of spatial and temporal analysis is only possible with the sequencing of the positive isolates and demonstrates the utility of sequencing for outbreak detection in a food process facility. The study shows the potential for *C. sakazakii* to persist for considerable periods in a dairy process facility. Other studies have also reported on the persistence of *C. sakazakii* in dairy facilities. Mullane et al. (2007) using pulse field electrophoresis reported that persistence of particular clones of *C. sakazakii* may persist in a PIF facility arising from a 1‐year monitoring study. Reich et al. (2010) studied the occurrence of *C. sakazakii* in a PIF but in the absence of using any molecular techniques to type the isolates recovered, they could make no comment on persistence in the process environment. Pei et al. (2019) used pulse field electrophoresis and MLST to study *C. sakazakii* occurrence in eight PIF process facilities in China and reported that *C. sakazakii* isolates with the same PFGE type and ST type were isolated over a 2‐year period. Compared to these prior studies that relied on PFGE and MLST, the application of WGS in the present work allowed for more precise identification of closely related isolates within the same sequence type, revealing finer genetic clustering and longer term persistence patterns that PFGE or MLST alone might not distinguish. The use of whole‐genome sequencing in the current work presents more precision in terms of identifying similar genetic clusters and, in our knowledge, represents the first time that sequencing has been used to track and identify persistence of *C. sakazakii* in a dairy process environment. However, the source of the persistent contamination observed in this study remains unclear. It could originate from a common niche within equipment that was inadequately cleaned, or alternatively from repeated introduction via raw materials or other external sources. Further targeted sampling of specific equipment surfaces and incoming materials would be required to distinguish between these possibilities.

Despite the insights provided by this study, several limitations should be acknowledged. Sampling was conducted on a monthly basis, which may have missed short‐term peaks of contamination that could occur and resolve between sampling events. Additionally, no environmental parameters such as humidity, temperature, or airflow were systematically monitored or correlated with detection events, limiting the ability to identify potential environmental drivers of persistence. Future studies incorporating more frequent sampling and environmental monitoring would help address these gaps.

## 5 Conclusion

This study successfully undertakes a yearlong surveillance program for *Cronobacter* spp. in the environment of a large‐scale Irish milk protein concentrate processing facility. Sequencing and species identification confirmed that all positive isolates were *C. sakazakii* strains. MLST analysis indicated several sequence types among the positive isolates. Phylogenetic analysis coupled with temporal and spatial analysis of the isolates indicated a significant amount of persistence of the same strain for periods of time of at least corresponding to the surveillance period (1 year). WGS proved to be a highly effective and precise fingerprinting tool, offering superior resolution compared to traditional methods such as PFGE and MLST for tracking long‐term contamination and persistence of specific *C. sakazakii* strains over extended periods. Overall, the study demonstrated the utility of using WGS as an additional fingerprinting tool in surveillance work for pathogens such as *C. sakazakii* in dairy processing facilities.

These findings highlight the importance of implementing proactive and ongoing hygiene monitoring programs in dairy powder processing facilities, including regular environmental sampling combined with advanced molecular tools like WGS. Such approaches can enable early detection of persistent strains, facilitate targeted cleaning interventions, and ultimately reduce the risk of product contamination.

## Author Contributions

**Qicheng Hao:** conceptualization, methodology, software, data curation, investigation, validation, formal analysis, writing – original draft, writing – review and editing. **Francis Butler:** conceptualization, methodology, writing – review and editing, student supervision.

## Funding

This work was supported by University College Dublin (UCD), Ireland; the Dairy Processing Technology Centre (DPTC), Ireland; and the China Scholarship Council (CSC) under the State Scholarship Fund.

## Conflicts of Interest

The authors declare no conflicts of interest.

## Supporting information


**Figure S1:** Phylogenetic tree for the *Cronobacter sakazakii* isolates recovered in the process facility (ST4 is shown in red).

## Data Availability

The data that support the findings of this study are available on request from the corresponding author. The data are not publicly available due to privacy or ethical restrictions.
