# Supplementary material for: Whole‐Genome Sequencing‐Based Monitoring of Cronobacter sakazakii ST4 Persistence in an Irish Dairy Powder Facility
Source: Food Sci Nutr. 2026 May 15;14(5):e71717. doi: 10.1002/fsn3.71717 (PMC13176939; doi:10.1002/fsn3.71717)
Supplement: Supplementary file 1 — Figure S1: Phylogenetic tree for the Cronobacter sakazakii isolates recovered in the process facility (ST4 is shown in red). [file FSN3-14-e71717-s001.docx]

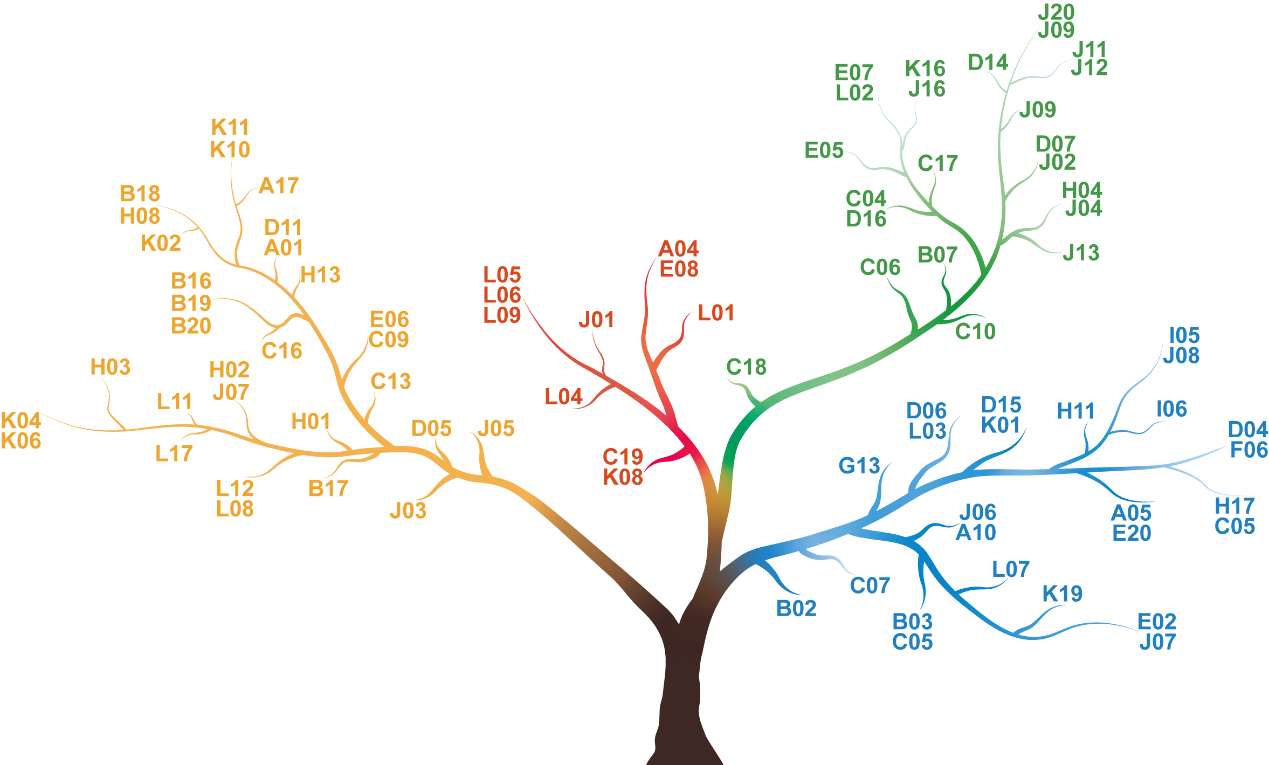


Supplementary Figure.S1 Phylogenetic tree for the *Cronobacter sakazakii* isolates recovered in the process facility (ST4 is shown in red)
